# Benzothiazinones Mediate Killing of *Corynebacterineae* by Blocking Decaprenyl Phosphate Recycling Involved in Cell Wall Biosynthesis*

**DOI:** 10.1074/jbc.M113.522623

**Published:** 2014-01-20

**Authors:** Shipra Grover, Luke J. Alderwick, Arun K. Mishra, Karin Krumbach, Jan Marienhagen, Lothar Eggeling, Apoorva Bhatt, Gurdyal S. Besra

**Affiliations:** From the ‡ School of Biosciences, Institute of Microbiology and Infection, University of Birmingham, Edgbaston, Birmingham B15 2TT, United Kingdom and; the § Institute for Bio and Geosciences Biotechnology (IBG-1), Research Centre Juelich, D-52425 Juelich, Germany

**Keywords:** Bacterial Metabolism, Carbohydrate Metabolism, Cell Wall, Drug Resistance, Polysaccharide

## Abstract

Benzothiazinones (BTZs) are a new class of sulfur containing heterocyclic compounds that target DprE1, an oxidoreductase involved in the epimerization of decaprenyl-phosphoribose (DPR) to decaprenyl-phosphoarabinose (DPA) in the *Corynebacterineae*, such as *Corynebacterium glutamicum* and *Mycobacterium tuberculosis*. As a result, BTZ inhibition leads to inhibition of cell wall arabinan biosynthesis. Previous studies have demonstrated the essentiality of *dprE1*. In contrast, Cg-UbiA a ribosyltransferase, which catalyzes the first step of DPR biosynthesis prior to DprE1, when genetically disrupted, produced a viable mutant, suggesting that although BTZ biochemically targets DprE1, killing also occurs through chemical synthetic lethality, presumably through the lack of decaprenyl phosphate recycling. To test this hypothesis, a derivative of BTZ, BTZ043, was examined in detail against *C. glutamicum* and *C. glutamicum*::*ubiA*. The wild type strain was sensitive to BTZ043; however, *C. glutamicum*::*ubiA* was found to be resistant, despite possessing a functional DprE1. When the gene encoding *C. glutamicum Z*-decaprenyl-diphosphate synthase (*NCgl2203*) was overexpressed in wild type *C. glutamicum,* resistance to BTZ043 was further increased. This data demonstrates that in the presence of BTZ, the bacilli accumulate DPR and fail to recycle decaprenyl phosphate, which results in the depletion of decaprenyl phosphate and ultimately leads to cell death.

## Teaser

**Background:** Benzothiazinones inhibit cell wall arabinan biosynthesis, which is lethal for *Corynebacterineae.*

**Results:** *Corynebacteria* can evade the action of benzothiazinones in the absence of decaprenyl phosphoribose synthesis by increasing the intracellular decaprenyl phosphate pool.

**Conclusion:** Benzothiazinones induce synthetic lethality in *Corynebacterineae* by blocking decaprenyl phosphate recycling.

**Significance:** Increased production of decaprenyl phosphate is a new mechanism of resistance to benzothiazinones.

## Introduction

Tuberculosis (TB)^3^ remains the single most important bacterial cause of mortality and morbidity globally, causing 8.7 million new cases and 1.4 million deaths in 2011 (WHO, 2011). Directly observed treatment, short course, a mixture of anti-TB drugs comprised of pyrazinamide, isoniazid, ethambutol, and rifampicin, has been used successfully throughout the last three decades as a treatment regimen for patients suffering from TB. However, the emergence of multidrug resistant (MDR), extensively drug-resistant (XDR), and now totally drug-resistant strains of *Mycobacterium tuberculosis* means that the need for new drugs, delivery systems, and regimes for improved treatment of TB is of paramount importance.

**FIGURE 1.**
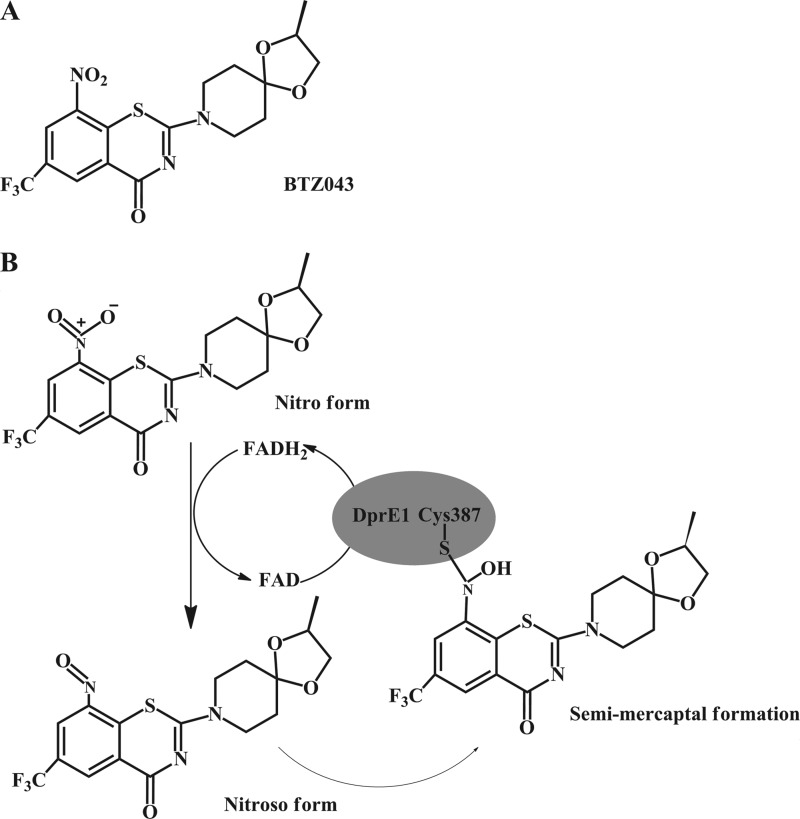
**Structure and mode of inhibition of BTZ043.** *A,* structure of the antitubercular compound BTZ043 that targets DprE1. *B,* BTZ043 is reduced into a nitroso-derivative by the flavin cofactor of DprE1 (36). This electrophilic nitroso-derivative irreversibly binds Cys-387 in the active site of DprE1 (14, 35, 36) and forms a semi-mercaptal adduct that renders the enzyme inactive.

The sulfur-containing benzothiazinones (BTZ) have recently been identified as a new class of highly potent anti-TB agents (Fig. 1*A*), which display very low minimum inhibitory concentrations (MICs) for *M. tuberculosis* (1–4 ng/ml) and fast-growing *Mycobacterium* species, such as *Mycobacterium smegmatis* (0.1–80 ng/ml) (1). Furthermore, BTZ exhibits high efficacy against MDR and XDR-TB strains in addition to being non-toxic toward human cell lines (2). Whole genome sequencing studies of spontaneous mutants resistant to BTZ combined with biochemical experiments established that BTZs target DprE1 (*Rv3790*), which is a FAD-containing oxidoreductase (Fig. 1*B*) that functions in concert with a NADH-dependent reductase DprE2 (*Rv379*1) to catalyze the epimerization of decaprenylphosphoryl-d-ribose (DPR) to decaprenylphosphoryl-d-arabinose (DPA) (1, 3). DPR is first oxidized to a keto intermediate, DPX via DprE1, and then reduced to DPA by DprE2. The essentiality of DprE1 has been established in *M. smegmatis* using a conditional mutant where the chromosomal copy of *dprE1* could be deleted only in the presence of a plasmid-encoded *dprE1* copy (4). Similarly, attempts to delete the *Corynebacterium glutamicum* orthologue, *NCgl0187*, have also been unsuccessful (5). Interestingly, the decaprenylphosphoribose-phosphate phosphatase (*Rv3807*) is not essential in *M. tuberculosis* (6) and redundancy is observed for DprE2 in *C. glutamicum* (5). The production of DPA and utilization in arabinan biosynthesis, as illustrated through genetic experiments and inactivation of *dprE1*, is therefore essential for the formation of a complete *M. tuberculosis* and *C. glutamiucm* cell wall (7).

The first step of the pathway for DPA biosynthesis is catalyzed by UbiA, a ribosyltransferase that generates decaprenylphosphoribose phosphate by condensation of phosphoribosyl pyrophosphate with decaprenyl phosphate, followed by dephosphorylation (*Rv3807*) affording DPR. However, unlike in *M. tuberculosis*, inactivation of *ubiA* in *C. glutamicum* produces a viable mutant that is unable to synthesize DPA and is devoid of cell wall arabinan, suggesting that synthesis of DPA and cell wall arabinan itself is not essential for survival in *C. glutamicum* (8, 9).

In this study, we examined the mode of action of BTZ inhibition on *C. glutamicum* and *C. glutamicum*::*ubiA*. The data shows that BTZ perturbs *C. glutamicum* growth but was ineffective against the Cg-UbiA mutant even though DprE1 was functionally intact. In addition, the inhibitory effect of BTZ on cell wall arabinan biosynthesis was examined when the *C. glutamicum Z*-decaprenyl-diphosphate synthase, encoded by *NCgl2203* (UppS), was overexpressed in *C. glutamicum*. The results demonstrate that overexpression of UppS was able to rescue the wild type strain (BTZ MIC 20 μg/ml) and shift the MIC of BTZ for this strain to >40 μg/ml. In conclusion, BTZ blocks the recycling of decaprenyl phosphate as in its presence, the cell continually synthesizes DPR, which is presumably toxic to the cells, and subsequently renders decaprenyl phosphate unavailable for the biosynthesis of other macromolecules, such as peptidoglycan, lipoarabinomannan, and arabinogalactan. However, the overexpression of a prenyl synthase supplements enough decaprenyl phosphate to aid in cell wall biosynthesis.

## EXPERIMENTAL PROCEDURES

### 

#### 

##### Chemicals, Reagents, and Enzymes

All chemicals and solvents were from Sigma, Bio-Rad, and Fisher Chemicals (UK), unless otherwise stated, and were of AnalR grade or equivalent. Plasmids were propagated during cloning in *Escherichia coli* Top10 cells (Invitrogen). All restriction enzymes and Phusion DNA polymerase enzyme were sourced from New England Biolabs. A Bioline quick ligation kit was used to perform ligation reactions. Oligonucleotides were from MWG Biotech Ltd. and PCR fragments were purified using the QIAquick gel extraction kit (Qiagen). Plasmid DNA was purified using the QIAprep purification kit (Qiagen).

##### Bacterial Strains and Growth Conditions

*E. coli* Top 10 cells were routinely grown in Luria-Bertani broth (LB, Difco) at 37 °C. *C. glutamicum* ATCC 13032, *C. glutamicum*-pVWEx2, and *C. glutamicum*-pVWEx2-*uppS* (10 μg/ml tetracycline) was grown on rich brain heart infusion medium (BHI, Difco) and *C. glutamicum*::*ubiA* (25 μg/ml of kanamycin) on BHI containing 9.1% sorbitol (BHIS) supplemented with appropriate antibiotics for selection.

##### Construction of Plasmids and Strains

*C. glutamicum-uppS (NCgl2203*) was cloned in pVWEx2 under the isopropyl β-d-thiogalactopyranoside-inducible Ptac promoter and was amplified from genomic DNA using the primers: 2203vwex, forward, 5′-GCATGTCTAGAAGGAGATATAGATGTGAGTGAATTCCAAGTA-3′ and 2203vwex, reverse, 5′-ATTAGGATCCTTATGCGCTTCCGAATCTGCG-3′ (restriction sites underlined). The PCR product was ligated into plasmid pVWEx2 using XbaI and BamHI restriction sites, yielding *C. glutamicum*-pVWEx2-*uppS*. The empty pVWEx2 and pVWEx2-*uppS* plasmids were electroporated into *C. glutamicum* using a standard protocol (10). The pVWEx2-*uppS* plasmid was subsequently sequenced by Eurofins MWG Operon. The *C. glutamicum*::*ubiA* strain was obtained as described previously (8).

##### Expression of uppS in C. glutamicum

*C. glutamicum*-pVWEx2-*uppS* cultures were grown overnight and then used to inoculate 50 ml of BHI (30 °C, 180 rpm, 10 μg/ml of tetracycline) and subsequently induced with 0.5 mm isopropyl β-d-thiogalactopyranoside at an *A*_600_ 0.6 and incubated at 30 °C for 24 h. Cells were harvested (15 min, 7000 × *g*), washed twice with phosphate-buffered saline, and resuspended in 50 mm MOPS (pH 7.9) containing lysozyme (0.2 mg/ml). The resuspended cell pellets were incubated at 37 °C for 1 h to allow cell lysis. The suspension was centrifuged (20 min, 27,000 × *g*, 20 min) and the supernatant checked for the presence of the Cg-UppS protein on SDS-PAGE gels.

##### Effect of BTZ043 on C. glutamicum Strains

To determine the *C. glutamicum* and *C. glutamicum* pVWEx2 MIC of BTZ043, ∼10^8^ cells were used to inoculate 2 ml of BHI broth containing 0 to 20 μg/ml of BTZ043 in a stepwise gradient and the *A*_600_ measured up to 48 h. To determine the effect of increasing concentrations of BTZ043 on *C. glutamicum*::*ubiA* and *C.glutamicum*-pVWEx2-*uppS,* ∼10^8^ cells were used (30 °C, BHIS, 200 rpm) to inoculate 2 ml of BHIS containing 0 and 20 μg/ml of BTZ043 and the *A*_600_ measured up to 48 h. The cultures were also grown for 48 h as above and the viability count was determined by spotting 10 μl of serially diluted cultures (up to dilution 10^−8^) on BHIS plates for *C. glutamicum*::*ubiA* and *C. glutamicum*-pVWEx2-*uppS.* The MIC was defined as the minimal concentration required to completely inhibit 99% of the growth.

##### BTZ043 Inhibits DPA Synthesis in C. glutamicum Strains

To characterize the effect of BTZ043 on DprE1, *p*-[^14^C]Rpp was synthesized as described previously (11) and supplemented as a substrate for the *in vitro* synthesis of DPA. Accumulation of DPR in the presence of BTZ043 was chosen as a parameter to monitor the effect of BTZ043. Cell membranes from *C. glutamicum* and *C. glutamicum*::*ubiA* were prepared as described previously and assayed for DPA biosynthesis (12). Decaprenol phosphate (50 μg, 5 mg/ml stored in ethanol, 1 μl) was dried under nitrogen and resuspended in buffer A (50 mm MOPS, 10 mm MgCl_2_, pH 8.0) and sonicated. The basic assay mixture consisted of 400 μg of membranes and a P60 fraction (13), 25 mm ATP, 25 mm FAD, 25 mm NAD, 25 mm NADP, and BTZ043 (20 μg/ml in DMSO) in a final volume of 80 μl of buffer A and initiated by the addition of 65,000 cpm of *p*-[^14^C]Rpp. Reactions were incubated at 30 °C for 1 h and quenched by the addition of 4 ml of CHCl_3_/CH_3_OH/H_2_O (10:10:3, v/v) and mixed for 15 min. The assay mixture was combined with 1.75 ml of CHCl_3_ and 0.75 ml of H_2_O, mixed for 15 min, and centrifuged at 3000 × *g* for 10 min. The lower organic phase was removed and washed twice with 2 ml of CHCl_3_/CH_3_OH/H_2_O (3:47:48, v/v), centrifuged at 3000 × *g* for 15 min, recovered, and dried under nitrogen. The resulting products were resuspended in 20 μl of CHCl_3_/CH_3_OH (2:1, v/v) and an aliquot was subjected to scintillation counting using 5 ml of fluid, and a second aliquot was subjected to TLC analysis using silica gel plates (5735 Silica Gel 60F254, Merck) developed in CHCl_3_/CH_3_OH/H_2_O/NH_4_OH/CH_3_COONH_4_ (180:140:23:9:9, v/v), and visualized by phosphorimaging by exposing the TLCs to a phosphorimaging screen (Kodak) for 24 h.

##### DprE1 Is Functional in C. glutamicum and C. glutamicum::ubiA

To characterize the effect of BTZ043 on DprE1, DP[^14^C]R was supplemented as a substrate for the *in vitro* synthesis of DP[^14^C]A (11). Cell membranes from *C. glutamicum* and *C. glutamicum*::*ubiA* were assayed for DPA biosynthesis activity as described previously. DP[^14^C]R was prepared using *p*-[^14^C]Rpp as described previously (11). Briefly, to prevent DPA formation, BTZ043 (20 μg/ml in DMSO) was added to the assay in a final volume of 80 μl (including buffer A, co-factor mixture, membranes, and P60). The resulting DP[^14^C]R was extracted as described previously and dried under nitrogen (14). The resulting assay products were resuspended in 20 μl of CHCl_3_/CH_3_OH (2:1, v/v) and an aliquot was subjected to TLC analysis using silica gel plates (5735 Silica Gel 60F254, Merck) developed in CHCl_3_/CH_3_OH/H_2_O/NH_4_OH/CH_3_COONH_4_ (180:140:23:9:9, v/v) and visualized by autoradiography by exposure of TLCs to x-ray film (Kodak X-Omat). The DP[^14^C]R thus prepared was used as a substrate for determining the functionality of DprE1 in *C. glutamicum* and *C. glutamicum*::*ubiA.* The basic assay mixture consisted of DP[^14^C]R (20,000 cpm) dried and resuspended in buffer A, 500 μg of membranes, and P60 fraction, co-factor mixture and BTZ043 (20 μg/ml in DMSO) in a final volume of 80 μl of buffer A. Reactions were incubated at 30 °C for 1 h and quenched by the addition of 4 ml of CHCl_3_/CH_3_OH/H_2_O (10:10:3, v/v), and mixed for 15 min. The assay mixture was combined with 1.75 ml of CHCl_3_ and 0.75 ml of H_2_O, mixed for 15 min, and centrifuged at 3,000 × *g* for 10 min. The lower organic phase was removed and washed twice with 2 ml of CHCl_3_/CH_3_OH/H_2_O (3:47:48, v/v), centrifuged at 3,000 × *g* for 15 min, recovered, and dried under nitrogen. The resulting residue was resuspended in 20 μl of CHCl_3_/CH_3_OH (2:1, v/v) and equal counts (10,000 cpm) were subjected to TLC analysis using silica gel plates (5735 Silica Gel 60F254, Merck) developed in CHCl_3_/CH_3_OH/H_2_O/NH_4_OH/CH_3_COONH_4_ (180:140:23:9:9, v/v) and visualized by phosphorimaging by exposing the TLCs to a phosphorscreen (Kodak) for 24 h.

##### Increased Synthesis of Decaprenyl Phosphate Occurs in C. glutamicum-pVWEx2-uppS

To characterize the effect of overexpression of the *C. glutamicum* decaprenyl-phosphate synthase (UppS) on decaprenyl phosphate synthesis, [4-^14^C]isopentenyl pyrophosphate (IPP), and triammonium salt (40–60 mCi/mmol, PerkinElmer Life Sciences) was supplemented as a substrate. Cell membranes from *C. glutamicum*-pVWEx2 and *C. glutamicum*-pVWEx2-*uppS* were prepared as described previously (12) and assayed for decaprenyl phosphate biosynthesis activity.

[^14^C]IPP (30 μm stored in ethanol) was dried under nitrogen and resuspended in buffer A with 400 μg of membranes, 100 μm geranyl diphosphate, 100 μm dimethyl allyl diphosphate, and BTZ043 (20 μg/ml in DMSO), where required in a final volume of 55 μl of buffer A. Reactions were incubated at 30 °C for 1 h and quenched by the addition of 1 ml of water-saturated NaCl and extracted using butanol saturated with water. The butanol fraction was dried under nitrogen and subjected to mild acid hydrolysis using 0.1 n HCl in 50% ethanol at 37 °C for 24 h. The radiolabeled dephosphorylated products were recovered by addition of an equal volume of chloroform. The extracts were washed twice with water and the organic phase was recovered, dried, and resuspended in 20 μl of CHCl_3_/CH_3_OH (2:1, v/v). An aliquot was taken for liquid scintillation counting and equal counts (20,000 cpm) were subjected to TLC analysis using reverse phase silica gel plates (Silica Gel 60 RP-18 F254S, Merck) developed in methanol/acetone (8:2, v/v) and visualized by autoradiography by exposing the TLCs to x-ray film (Kodak X-Omat) for 24 h and compared with known standards using ethanolic molybdophosphoric acid and plates heated gently using a heat-gun.

##### Analysis of AG and LAM Biosynthesis in C. glutamicum-pVWEx2 and C. glutamicum-pVWEx2-uppS Treated with BTZ043

To analyze the effect of overexpression of decaprenyl-phosphate synthase on the cell wall component AG in *C. glutamicum,* overnight cultures of *C. glutamicum*-pVWEx2 and *C. glutamicum*-pVWEx2-*uppS* were used to subculture 20 ml of BHI media and grown to *A*_600_ 0.4–0.6. The cultures were induced with 0.5 mm isopropyl β-d-thiogalactopyranoside and half of the culture was treated with BTZ043 at 0.75× MIC (15 μg/ml) for 2 h. Both treated and untreated cultures were labeled with 1.0 μCi/ml of [^14^C]glucose (250–360 mCi/mmol, PerkinElmer Life Sciences) and grown for 48 h (30 °C, 200 rpm). The mycolylarabinogalactan complex was isolated and sugar hydrolysis was performed on all the samples as previously described (15). The radioactive sugar samples were analyzed by loading equal counts (20,000 cpm) on HPTLC-cellulose plates developed three times in formic acid/water/tertiary butanol/methylethyl ketone (3:3:8:6) and visualized by autoradiography by exposing the TLC to x-ray film (Kodak X-Omat) for 4 days.

To analyze the effect of overexpression of decaprenyl-phosphate synthase on the cell wall component LAM/LM in *C. glutamicum,* overnight cultures of *C. glutamicum*-pVWEx2 and *C. glutamicum*-pVWEx2-*uppS* were used to subculture 40 ml of BHI media and grown to *A*_600_ 0.4–0.6. The cultures were induced with 0.5 mm isopropyl β-d-thiogalactopyranoside and labeled with 1.0 μCi/ml of [^14^C]glucose (250–360 mCi/mmol, PerkinElmer Life Sciences) and grown for 1 h (30 °C, 200 rpm). At time 0 h, the culture was split into 5-ml aliquots and BTZ043 (20 μg/ml) was introduced into half of the aliquots to afford treated and untreated cultures. The cultures were then sampled every 2 h by using 5-ml aliquots, centrifuged, and snap frozen using liquid nitrogen. LAM/LM analysis was performed on all the samples as previously described (8). The [^14^C]LAM/LM samples were analyzed by loading equal counts (3000 cpm) on SDS-PAGE gel and visualized by autoradiography by exposing the gels to x-ray film (Kodak X-Omat) for 14 days.

## RESULTS

### 

#### 

##### Effect of BTZ043 on C. glutamicum Wild Type and C. glutamicum::ubiA

**FIGURE 2.**
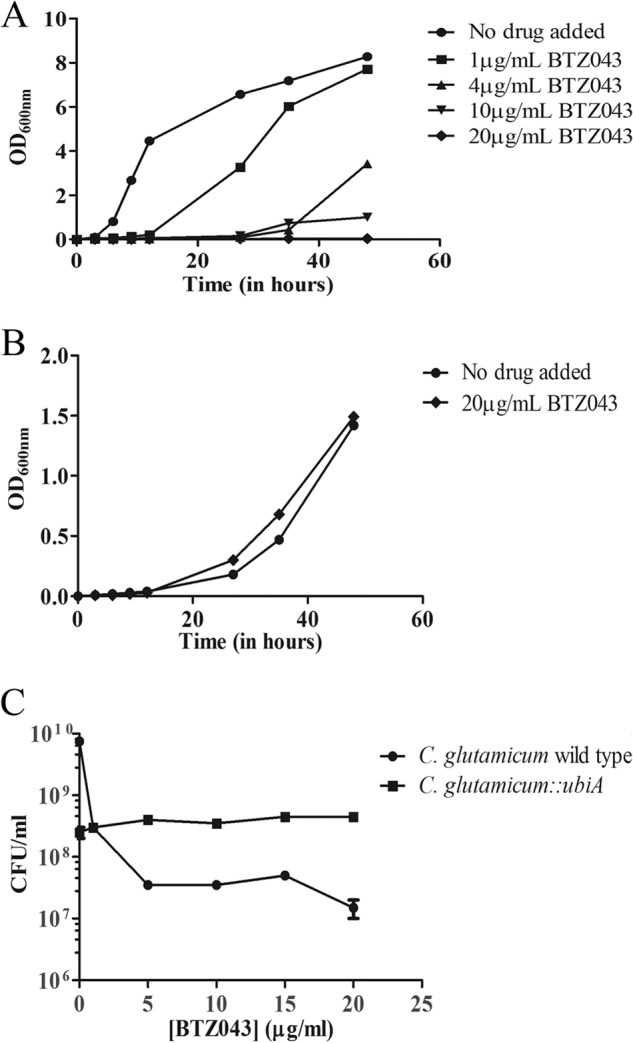
**Comparison of growth, viability, and susceptibility to BTZ043 of *C. glutamicum* and *C. glutamicum*::*ubiA*.** *A, C. glutamicum* was examined for its susceptibility to BTZ043 in liquid media at a range of concentrations (0, 1, 4, 10, and 20 μg/ml). *B, C. glutamicum*::*ubiA* was examined for its susceptibility to BTZ043 in liquid media at a final concentration of 20 μg/ml (MIC for *C. glutamicum*). *C,* the cell viability of *C. glutamicum* and *C. glutamicum*::*ubiA* was tested at a range of BTZ043 concentrations (0, 1, 5, 10, 15, and 20 μg/ml) by spotting ∼10^8^-10^9^ cells on BHIS-agar plates incubated at 30 °C for 48 h after treatment with 20 μg/ml of BTZ043.

The BTZ analog BTZ043 targets DprE1, which is a FAD-containing oxidoreductase conserved across the *Corynebacterineae* (14). Sequence alignment analysis of *Rv3790* and *NCgl0187* (the open reading frames encoding DprE1 from *M. tuberculosis and C. glutamicum*, respectively) shows that the amino acid sequences of these two orthologs are 65% identical (5). Furthermore, *Cg*-DprE1 also contains all the conserved active site residues, including Cys-414, which corresponds to Cys-387 in *Mt*-DprE1 that forms a covalent semi-mercaptal linkage with the activated nitroso group of BTZ043 (Fig. 1*B*). *C. glutamicum* and *M. tuberculosis* share very similar cell wall architecture and due to its genetically tractable genome, we have used *C. glutamicum* extensively as an excellent model organism to study the molecular genetics of mycobacterial cell wall biosynthesis (8, 16, 17). *C. glutamicum*::*ubiA* is a particularly interesting genetically modified strain, in as much as it is completely devoid of arabinan in its cell wall. UbiA encodes for a decaprenylphosphoryl-5-phospho-β-d-ribose synthase and is non-essential in *C. glutamicum.* Because the primary action of BTZ043 is reportedly due to the blockage of cell wall arabinan biosynthesis, we cultured *C. glutamicum* (wild type) and *C. glutamicum*::*ubiA* in liquid media over a 48-h period and examined their susceptibility to BTZ043 at a range of concentrations (Fig. 2, *A* and *B*). We determined that the MIC of BTZ043 against wild type *C. glutamicum* is 20 μg/ml (Fig. 2*A*). Although *C. glutamicum*::*ubiA* is inherently slow growing, when we repeated the experiment with this mutant, we observed that BTZ043 failed to inhibit the growth of this arabinan-deficient mutant at a concentration corresponding to the MIC of the wild type strain (Fig. 2*B*). Furthermore, we conducted cell viability assays against *C. glutamicum* and *C. glutamicum*::*ubiA* against increasing concentrations of BTZ043; our data demonstrates that BTZ043 elicits a 1000-fold decrease in cell viability against *C. glutamicum* in comparison to *C. glutamicum*::*ubiA* in which BTZ043 failed to inhibit its growth with no observable effect on cell viability (Fig. 2*C*). Therefore, *C. glutamicum*::*ubiA* was determined to be resistant to BTZ043, in comparison with the sensitive wild type *C. glutamicum* strain.

##### BTZ043 Inhibits DPA Synthesis in C. glutamicum and C. glutamicum::ubiA with a Functional DprE1

**FIGURE 3.**
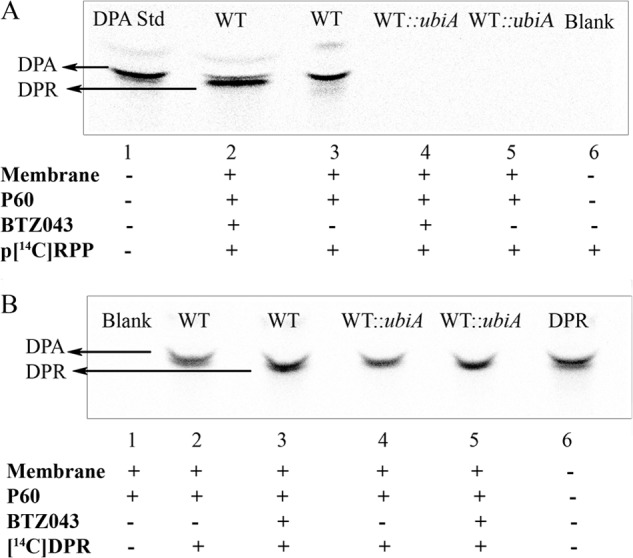
**DprE1 is functionally active in *C. glutamicum* and *C. glutamicum*::*ubiA*.** Membrane and “P60” extracts were prepared from *C. glutamicum* and *C. glutamicum*::*ubiA* and examined for their ability to generate DP[^14^C]A from exogenously supplied *p*-[^14^C]Rpp (*A*) and DP[^14^C]R (*B*) in the absence and presence of BTZ043 (20 μg/ml). *A,* membrane and P60 extracts from *C. glutamicum* are capable of synthesizing DP[^14^C]A when supplied with *p*-[^14^C]Rpp and were inhibited in assays that included BTZ043, whereas no ^14^C-labeled products were seen in preparations from *C. glutamicum*::*ubiA* upon treatment with BTZ043. *B,* DprE1 activity was directly assessed by supplementing DP[^14^C]R as a substrate. In the absence of BTZ043, both *C. glutamicum* and *C. glutamicum::ubiA* were able to produce DP[^14^C]A, whereas DP[^14^C]A formation was completely ablated when the assays were repeated in the presence of BTZ043, suggesting the presence of a fully functional DprE1 in *C. glutamicum*::*ubiA*.

We prepared membrane preparations from *C. glutamicum* and *C. glutamicum*::*ubiA*, which were subsequently examined for their ability to synthesize DPA in the absence and presence of BTZ043. Initial experiments used *p*-[^14^C]Rpp as an exogenous substrate with extracts to monitor the conversion of *p*-[^14^C]Rpp to DP[^14^C]R and DP[^14^C]A, which were evaluated using thin-layer chromatography (TLC) followed by phosphorimaging (Fig. 3*A*). Analysis of the ^14^C-labeled products by TLC clearly demonstrates that *C. glutamicum* is able to utilize *p*-[^14^C]Rpp as a substrate and synthesize DP[^14^C]A (Fig. 3*A*, *lane 3*). However, this conversion to DP[^14^C]A is inhibited when reaction mixtures are incubated with BTZ043, affording only DP[^14^C]R (Fig. 3*A*, *lane 2*). We also performed control experiments that included known standards (DPA, Fig. 3*A*, *lane 1*) and an enzyme blank (Fig. 3*A*, *lane 6*). However, no ^14^C-labeled products were seen when reactions were carried out using membranes prepared from *C. glutamiucm*::*ubiA* either in the absence or presence of BTZ043 (Fig. 3*A, lanes 4* and *5*). The inability of *C. glutamiucm*::*ubiA* to synthesize DPR/DPA is due to the absence of UbiA, which is a decaprenylphosphoryl-5-phospho-β-d-ribose synthase and is required for transfer of ribose 5-phosphate from *p*-[^14^C]Rpp to the lipid carrier decaprenyl monophosphate, and represents the first committed step to initiate synthesis of DPR/DPA (18).

To examine endogenous DprE1 activity directly in *C. glutamicum* and *C. glutamicum*::*ubiA*, DP[^14^C]R was exogenously supplied as a substrate in the absence and presence of BTZ043. Reaction products were extracted, separated by TLC, and subjected to analysis by phosphorimaging, which demonstrates that DP[^14^C]A is capable of being synthesized in cell-free assays from both *C. glutamicum* and *C. glutamicum*::*ubiA* strains (Fig. 3*B*, *lanes 2* and *4*). Furthermore, the synthesis of DPA is blocked in the presence of BTZ043, thus indicating that both *C. glutamicum* and *C. glutamicum*::*ubiA* express a functional copy of DprE1 and is targeted effectively by BTZ043 (Fig. 3*B, lanes 3* and *5*). The apparent lack of BTZ043 inhibition of *C. glutamiucm*::*ubiA* grown in culture is in stark contrast to the effect of BTZ043 on wild type *C. glutamicum*, and suggests that its mode of action is not due solely to DprE1 inhibition. This synthetic viable phenotype is manifested by the interruption of *ubiA* in *C. glutamicum*, which serves to alleviate sensitivity toward BTZ043. Because UbiA signifies the first committed step in the biosynthetic pathway leading toward DPA formation, we hypothesized that the primary effect of BTZ043 was to create a situation whereby exposure of *C. glutamicum* to the drug causes an accumulation of DPR, which is presumably toxic to the cells. Subsequently, this results in a failure by the cell to recycle decaprenyl phosphate and its limited availability causes a severe growth phenotype in *Corynebacterineae*. This BTZ043-induced chemical synthetic lethality is removed in *C. glutamicum*::*ubiA* because decaprenylphosphoribose phosphate is not being produced as a precursor to DPR formation, thus it allows the recycling of decaprenyl phosphate to occur unhindered, which is required for cell wall peptidoglycan biosynthesis.

##### Overexpression of Cg-uppS, a Prenylphosphate Synthase Protects C. glutamicum from Inhibition by BTZ043

**FIGURE 4.**
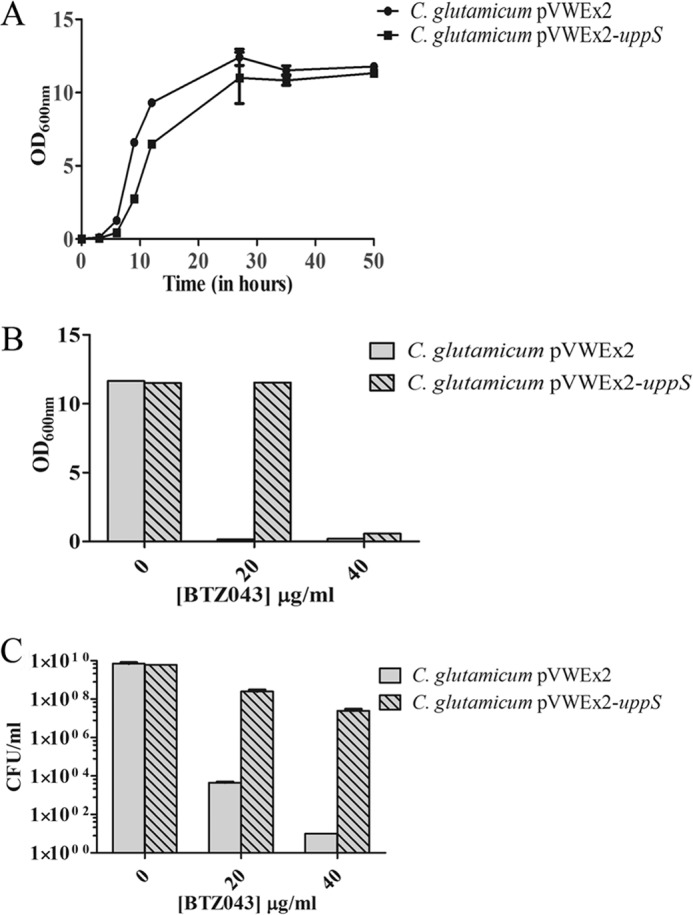
**Effect of BTZ043 treatment on *C. glutamicum*-pVWEx2 and *C. glutamicum*-pVWEx2-*uppS*.** The growth and viability of *C. glutamicum*-pVWEx2 and *C. glutamicum*-pVWEx2-*uppS* was examined in the presence of BTZ043. *A,* the growth rates of both *C. glutamicum* and *C. glutamicum*-pVWEx2-*uppS* were monitored in the absence of BTZ043 in liquid media for 48 h to check the effect of overexpression. *B,* the growth of *C. glutamicum*-pVWEx2 and *C. glutamicum*-pVWEx2-*uppS* was measured in the presence of BTZ043 at 1× MIC (20 μg/ml) and 2× MIC (40 μg/ml). The optical density (*A*_600_) obtained for *C. glutamicum*-pVWEx2-*uppS* was higher than *C. glutamicum*-pVWEx2 at both 1× MIC and 2× MIC, indicating increased tolerance to BTZ043. *C,* cell viability counts were determined for *C. glutamicum*-pVWEx2 and *C. glutamicum*-pVWEx2-*uppS* treated for 48 h at 1× and 2× MIC of BTZ043 after plating on BHI-agar plates.

The above data suggests that a fine balance exists in terms of the supply of decaprenyl phosphate for the biosynthetic pathways leading to DPA and lipid II formation, which ultimately result in the formation of AG and peptidoglycan, respectively. To further investigate the role of decaprenyl phosphate in cell wall biosynthesis, we overexpressed *uppS* (*NCgl2203*) (which encodes for a decaprenylphosphate synthase) by transforming *C. glutamicum* with pVWEx2-*uppS*. The growth and viability of both *C. glutamicum*-pVWEx2 and *C. glutamicum*-pVWEx2-*uppS* was examined following exposure to BTZ043 in liquid media, with both strains exhibiting comparable growth kinetics (Fig. 4*A*). After culturing *C. glutamicum*-pVWEx2 and *C. glutamicum*-pVWEx2-*uppS* in liquid media for 48 h in the absence and presence (1× and 2× MIC) of BTZ043, we measured end point readings for optical density and cell viability (Fig. 4, *B* and *C*). As expected BTZ043 inhibited the growth of *C. glutamicum*-pVWEx2 at concentrations of 1× and 2× MIC, whereas *C. glutamicum*-pVWEx2-*uppS* displayed a significantly increased resistance to BTZ043 with a MIC >40 μg/ml (Fig. 4*B*). Furthermore, the viable counts for *C. glutamicum*-pVWEx2-*uppS* were significantly higher than *C. glutamicum*-pVWEx2 at the 1× MIC (20 μg/ml) and 2× MIC (40 μg/ml) of BTZ043, respectively (Fig. 4*C*). The results demonstrated that Cg-UppS prenylphosphate synthase, when overexpressed, could rescue *C. glutamicum*-pVWEx2 from inhibition by BTZ043.

##### UppS Overexpression Results in an Increased Synthesis of Decaprenyl Phosphate in C. glutamicum

**FIGURE 5.**
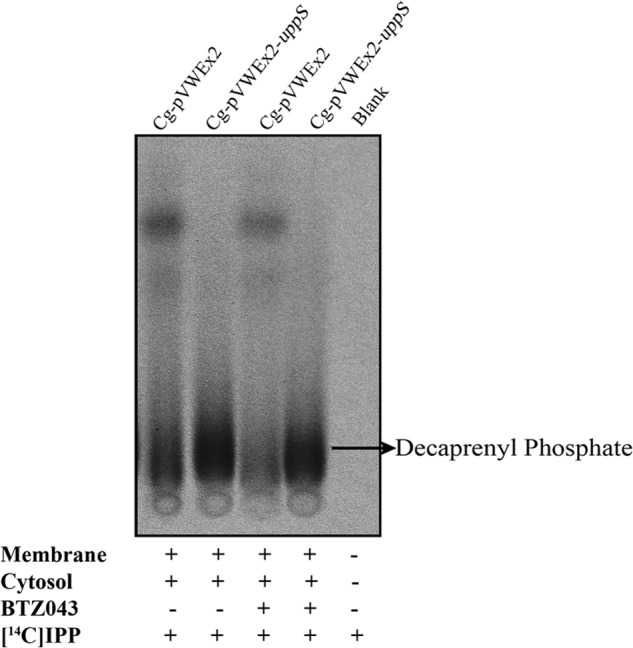
**Overexpression of *uppS* in *C. glutamicum* elicits an increase in decaprenyl phosphate synthesis.** Cell-free extracts prepared from *C. glutamicum*-pVWEx2 (*Cg-pVWEx2*) and *C. glutamicum*-pVWEx2-*uppS* (*Cg-pVWEx2-uppS*) were examined for any increased biosynthesis of decaprenyl phosphate in the presence and absence of BTZ043 (20 μg/ml), by labeling the assay mixtures with exogenous [^14^C]IPP. Assays were quenched after a 1-h incubation at 30 °C and reaction products were extracted with organic solvent. Equal counts (20,000 cpm) were loaded onto a reverse phase silica gel plates (Silica Gel 60 RP-18 F254S, Merck) developed in methanol/acetone (8:2, v/v) and visualized by phosphorimaging.

We prepared membrane extracts from *C. glutamicum*-pVWEx2 and *C. glutamicum*-pVWEx2-*uppS*, which were subsequently examined for their ability to synthesize decaprenyl phosphate in the absence and presence of BTZ043. We made use of [^14^C]IPP as an exogenous substrate supplied with membrane and cytosolic extracts to monitor the conversion of [^14^C]IPP to [^14^C]decaprenyl phosphate, which was evaluated using TLC followed by phosphorimaging (Fig. 5). Analysis of the ^14^C-labeled products by TLC clearly demonstrates that *C. glutamicum* is able to utilize [^14^C]IPP as a substrate and synthesize [^14^C]decaprenyl phosphate (Fig. 5). We conducted a densitometric analysis of the bands migrating on the TLC that correspond to decaprenyl phosphate and then calculated the apparent and actual cpm incorporated into [^14^C]decaprenyl phosphate from a total of 20,000 cpm loaded per reaction. *C. glutamicum* produced 481 ± 72 cpm/μg of protein, whereas overexpression of *uppS* in *C. glutamicum* increases the amount of [^14^C]decaprenyl phosphate by 77% (853 ± 16 cpm/μg of protein) and is illustrated by the increase in density of the band migrating on a TLC, corresponding to a standard decaprenyl phosphate (Fig. 5). When assays were supplemented with 20 μg/ml of BTZ043, synthesis of the decaprenyl phosphate pool appears to reduce in wild type *C. glutamicum*-pVWEx2 and remain relatively unchanged in *C. glutamicum*-pVWEx2-*uppS*, when compared with assays conducted in the absence of BTZ043 (Fig. 5). After normalizing the cpm for the absolute total counts recovered in each of the BTZ043-treated assays, we see a small, yet significant increase in the synthesis of decaprenyl phosphate, which equates to 8 and 10% for *C. glutamicum*-pVWEx2 and *C. glutamicum*-pVWEx2-*uppS*, respectively. Our data suggests that BTZ043 inhibition of endogenous DprE1 leads to a blockage in the DPA biosynthetic pathway, which prevents recycling of decaprenyl phosphate back into the cellular pool for the formation of decaprenyl phosphate-linked intermediates required for cell wall biosynthesis.

##### Overexpression of UppS Restores Arabinogalactan Biosynthesis in C. glutamicum Treated with BTZ043

**FIGURE 6.**
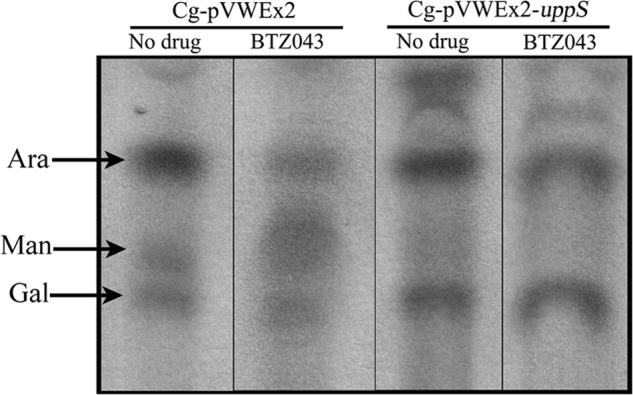
**Effect of BTZ043 on AG biosynthesis in *C. glutamicum*-pVWEx2 and *C. glutamicum*-pVWEx2-*uppS*.** The radioactive mAGP extracts were prepared from treated and untreated cultures of *C. glutamicum*-pVWEx2 (*Cg-pVWEx2*) and *C. glutamicum*-pVWEx2-*uppS* (*Cg-pVWEx2-uppS*) after 48 h of treatment with 0.75× MIC BTZ043 (15 μg/ml) and analyzed for their sugar content. The [^14^C]arabinose content decreased in Cg-pVWEx2 treated with BTZ043, whereas no decrease in [^14^C]arabinose was evident in BTZ043-treated Cg-pVWEx2-*uppS.* The *lanes* have been grouped from different parts of the same TLC exposure.

To further assess the effect of BTZ043 on incorporation of arabinose into the cell wall, *C. glutamicum*-pVWEx2 and *C. glutamicum*-pVWEx2-*uppS* were examined for their ability to synthesize AG when treated with BTZ043 (Fig. 6). The mycolylarabinogalactan peoptidoglycan complex (mAGP) was prepared from samples collected at 48 h from BTZ043 (15 μg/ml) treated and non-treated cultures and subsequently analyzed for its sugar composition. The distribution of radioactivity was detected between [^14^C]arabinose, [^14^C]mannose, and [^14^C]galactose after separation by TLC followed by autoradiography. Chromatographic analysis showed that in *C. glutamicum*-pVWEx2 treated with BTZ043 (15 μg/ml), incorporation of arabinose in AG is significantly reduced, whereas the level of arabinose being incorporated in *C. glutamicum*-pVWEx2-*uppS* remains relatively unchanged upon exposure to BTZ043 (15 μg/ml). TLC analysis of our hydrolyzed cell wall preparations also showed the presence of mannose, which we aportion to being an impurity coming from LM and LAM contaminants. We observe no notable difference in the galactan levels, which remain unchanged when we compare *C. glutamicum*-pVWEx2 and *C. glutamicum*-pVWEx2-*uppS* either treated or untreated with BTZ043 (Fig. 6). Thus, *C. glutamicum*-pVWEx2-*uppS* is able to synthesize AG unhindered in the presence of BTZ043 at 0.75× MIC.

##### Overexpression of UppS Restores LAM Biosynthesis in C. glutamicum Treated with BTZ043

**FIGURE 7.**
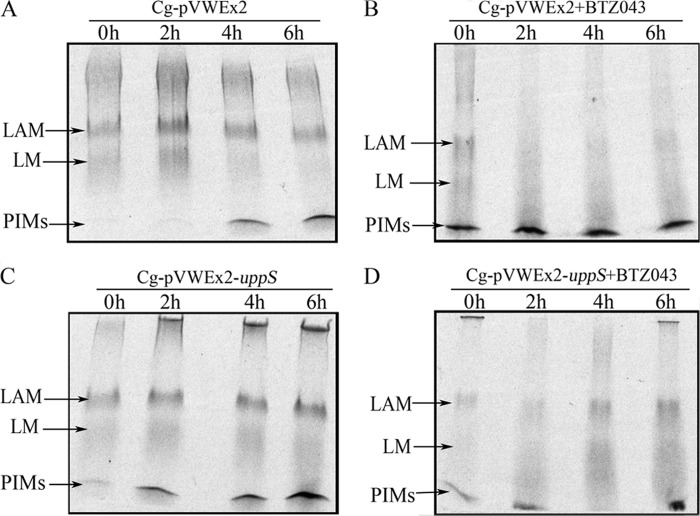
**SDS-PAGE analysis of lipoglycans extracted from *C. glutamicum*-pVWEx2(Cg-pVWEx2) (*A*), Cg-pVWEx2 treated with BTZ043 (*B*), *C. glutamicum*-pVWEx2-*uppS* (Cg-pVWEx2-*uppS*) (*C*), and Cg-pVWEx2-*uppS* treated with BTZ043 (*D*).** Cultures were grown in liquid media to *A*_600_ of 0.5 before the addition of [^14^C]glucose and BTZ043 (20 μg/ml) and growth was continued for up to 6 h.

*C. glutamicum*-pVWEx2 and *C. glutamicum*-pVWEx2-*uppS* were examined for their ability to synthesize LAM when treated with BTZ043 (20 μg/ml) (Fig. 7). Exponentially growing cultures of *C. glutamicum*-pVWEx2 and *C. glutamicum*-pVWEx2-*uppS* were treated with BTZ043 and samples were removed every 2 h from BTZ043-treated cultures (as well as non-treated cultures). Following lipoglycan extraction, the presence of [^14^C]LAM was detected in SDS-PAGE gels by autoradiography (Fig. 7). The SDS-PAGE analysis showed that in *C. glutamicum*-pVWEx2 cultures treated with BTZ043 (20 μg/ml), production of LAM and LM is significantly reduced over time when compared with untreated cultures of *C. glutamicum*-pVWEx2 (Fig. 7, *A* and *B*). However, modest levels of LAM and LM are still being produced in *C. glutamicum*-pVWEx2-*uppS* upon exposure to BTZ043 (20 μg/ml), as shown at 6 h when compared with both *C. glutamicum*-pVWEx2 and *C. glutamicum*-pVWEx2-*uppS* treated with BTZ043 (Fig. 7, *C* and *D*). Thus, *C. glutamicum*-pVWEx2-*uppS* is able to continually synthesize LAM in the presence of BTZ013 at 2× MIC.

## DISCUSSION

**FIGURE 8.**
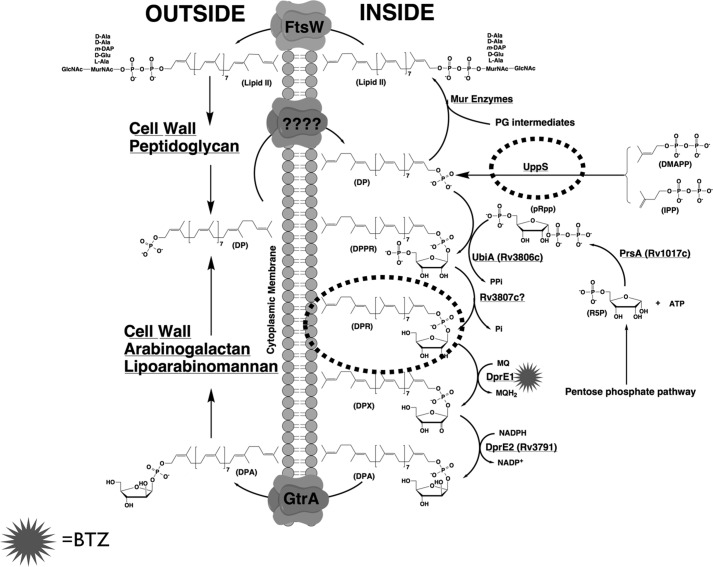
**A schematic representation of the proposed mechanism of action of BTZ on DprE1 inhibition and decaprenyl phosphate recycling.** DPA synthesis is crucial for biosynthesis of arabinan components of LAM and AG and begins with transfer of ribose phosphate from phosphoribosyl pyrophosphate (*pRPP*) to decaprenyl phosphate and follows the pathway where DprE1/E2 generates DPA from DPR. Treatment with BTZ043 (*star*) results in accumulation of DPR (highlighted in a *broken circle*) stalling LAM and AG biosynthesis and a block in decaprenyl phosphate recycling. Overexpression of UppS, the prenyl synthase (highlighted in a *broken circle*) causes restoration of the recycling pathway by supplementing enough decaprenyl phosphate for synthesis of cell wall components.

In 2009, it was reported that a new class of compounds called benzothiazinones exhibit extremely potent bactericidal activity against *M. tuberculosis*, as well as against drug-resistant clinical isolates, such as MDR-TB and XDR-TB strains (1). The most active compound, BTZ043, was shown by both biochemical and genetic experiments to target DprE1, a FAD-containing oxidoreductase responsible for the conversion of DPR to DPX (Fig. 8). Recently, the x-ray crystal structure of DprE1 in complex with BTZ043 was solved, highlighting the importance of a reactive Cys-387 residue in the active site, which forms a covalent semi-mercaptal linkage with the nitroso group of the molecule (Fig. 1*B*), thus providing the structural basis for suicide inhibition of DprE1 by BTZ (14, 19). DprE2 is a NADH-dependent reductase, which partners DprE1 as an “epimerization pair” that serves to reduce DPX to DPA (3). DPA is the sole substrate that is utilized by an array of membrane-embedded arabinofuranosyltransferases that are responsible for assembling the d-arabinan, which is an essential domain that is covalently attached to both LM and linear galactan, ultimately forming LAM and AG, respectively. EmbA, EmbB, and EmbC are three such arabinofuranosyltransferasess that are responsible, in part, for the biosynthesis of d-arabinan; each of these enzymes are targeted by ethambutol, a front-line drug currently in clinical use as part of the directly observed treatment, short course regimen (20, 21). However, due to the potency of BTZ043, DprE1 has been lauded by many within the mycobacterial research community as a “magic drug target.” Targeting d-arabinan biosynthesis ultimately results in the removal of covalent linkage between peptidoglycan and the outer mycolate layers, and is a salutary approach to development of new anti-mycobacterial agents. However, this is insufficient evidence to explain the vulnerable nature of DprE1 as a magic drug target, sitting at a critical intersection of cell wall biosynthesis. We sought to investigate the vulnerability of DprE1 as a drug target and the results from this study serve to deconvolute the observation that implicates DprE1 as being a particularly susceptible target within cell wall biosynthesis.

Because *M. tuberculosis* and *C. glutamicum* share a remarkably similar cell wall and many of the genes involved in cell wall biosynthesis are syntenic, *C. glutamicum* represents an excellent model organism for studying the molecular genetics associated with cell wall biosynthesis in *M. tuberculosis* (22–26). In this regard, we have successfully generated a panel of *C. glutamicum* mutant strains that incur lesions in cell wall arabinan due to the loss of genes that encode specific arabinofuranosyltransferasess or proteins involved in the processing of DPA. *C. glutamicum*::*ubiA* is a mutant strain that exhibits an extremely interesting phenotype, which is a complete loss of cell wall arabinan and covalently attached corynomycolates (8, 9, 27). This mutant becomes even more pertinent when we consider the fact that *C. glutamicum* is susceptible to the bactericidal activity of BTZ043, displaying an MIC of 20 μg/ml. The question must be asked, “why is *C. glutamicum*, a seemingly non-arabinan requiring member or the *Corynebacterineae*, susceptible to BTZ043, which has a reported mode of action as the blockage of d-arabinan formation by virtue of DprE1 inhibition?”

The data in this study does not exclude the possibility that killing results directly from toxicity associated with the accumulation of the DprE1 substrate DPR. Overproduction of Cg-UbiA could be toxic, either because of DP sequestration as we propose or through DPR accumulation. However, simultaneous overproduction of Cg-UppS and Cg-UbiA should either be lethal, if DPR accumulation is toxic, or rescue growth, if DP sequestration is actually killing the bacilli. We attempted to clarify this by amplifying Cg-*ubiA* from genomic DNA and cloning it into the inducible vector pEKEx2, which we have used successfully in previous studies on a number of membrane-embedded arabinosyltransferases (26, 28). We obtained a number of clones with the appropriate length, using a variety of PCR conditions and at least three different polymerases. However, upon sequencing different small mutations were present resulting in at least one non-acceptable amino acid change in our experiments. We then changed the cloning strategy by using another inducible vector pCLTON2, but again experienced similar non-acceptable mutations in Cg-*ubiA*. We therefore suggest that a weak expression even under non-inducing conditions is not tolerated when using an *E. coli* cloning host. We then attempted direct cloning in *C. glutamicum* but were again not successful in obtaining a correctly validated gene sequence. Altogether, this points to a delicate and tight control of Cg-*ubiA* expression, which is in line with the physiological data obtained in our studies.

However, our study does highlight the role of UppS, a decaprenyl-phosphate synthase, in rescuing *C. glutamicum* from the bactericidal effect of BTZ043 (Fig. 8). The implication here being that inhibition of DprE1 becomes acutely important when DPR begins accumulating in the cell membrane, and represents a metabolic “dead end” that halts the recycling of decaprenyl phosphate back into cell wall biosynthetic processes (Fig. 8). When UppS is overexpressed in *C. glutamicum*, the resultant phenotype is an increased tolerance to BTZ043 (Fig. 5). This increased tolerance can be attributed to the increased biosynthesis of decaprenyl phosphate, which is required primarily for the production of lipid II, an essential intermediate involved in peptidoglycan biosynthesis. This data is in accordance with our previous observations that although *C. glutamicum* has a cell wall with enough plasticity to dispense with d-arabinan, it still requires peptidoglycan to protect against the internal osmotic stress across the cytoplasmic membrane (15, 29). In this regard, a decrease in the expression of *uppS* from *Bacillus subtilis* has been shown to confer increased susceptibility to many late-acting cell wall antibiotics, such as β-lactams (30). However, this effect was significantly more pronounced when experiments were repeated using fosfomycin and d-cycloserine, which interfere with the early stages of peptidoglycan biosynthesis (30). In Gram-positive organisms, such as *B. subtilis* the undecaprenyl phosphate (C_55_-P) is the major polyprenyl carrier used in the synthesis of peptidogylcan as well as other cell wall components such as wall teichoic acids (31, 32). Furthermore, similar effects have been demonstrated in *Staphylococcus aureus*, whereby Targocil, an inhibitor of late-stage cell wall teichoic acid biosynthesis, causes an accumulation of undecaprenyl-linked intermediates, thus preventing recycling of the undecaprenyl-phosphate lipid carrier (33). Whereas lipid II is important for peptidoglycan biosynthesis, *Corynebacterineae*, such as *M. tuberculosis* and *C. glutamicum*, also use decaprenyl phosphate in the production of DPA and decaprenyl phosphomannose, which is directed toward the formation of d-arabinan and lipomannan (which is the precursor to LAM), respectively. We sought to investigate the effects of *uppS* overexpression on the biosynthesis of these cell wall polysaccharides when exposed to BTZ043. Our analysis of the total sugar content from mAGP isolated from *C. glutamicum*-pVWEx2 revealed an overall decrease in arabinose upon treatment with BTZ043. This result was expected because BTZ inhibits the production of the sole decaprenyl phosphate-based arabinose donor, DPA, which is required for biosynthesis of d-arabinan. However, we were able to counter this effect by overexpressing *uppS*, which resulted in a continued incorporation of [^14^C]arabinose into mAGP even up to BTZ043 concentrations that were 0.75× MIC (Fig. 6). Similarly, examination of the LAM pool from the BTZ043-treated *C. glutamicum*-pVWEx2 strain demonstrates a decrease in both LM and LAM, because both molecules employ decaprenyl phosphate-based sugar donors, in the form of decaprenyl phosphomannose and DPA. Because we observe a commensurate decrease in the formation of LM and LAM upon exposure to BTZ043, the notion that BTZ043 acts by inhibiting DPA formation is a false dichotomy. Indeed, overexpression of *uppS* restores the synthesis of both LM and LAM lipoglycan pools in the presence of BTZ043 (Fig. 7).

Mycobacteria can develop natural resistance to BTZs due to the occurrence of a single mutation in the operon coding for the Cys-387 residue in DprE1, which is substituted with an Ala or Ser, as observed in *Mycobacterium avium* and *Mycobacterium aurum*, respectively (1). BTZ043 functions as a pro-drug (34, 35) that requires reduction of the nitro group to an electrophilic nitroso-derivative to react with the Cys-387 residue in the active site of DprE1. This reaction is catalyzed either by DprE1 itself (36) or by other oxygen-insensitive nitroreductases (34). Bacterial nitroreducases, such as NfnB are capable of rendering the drug inactive by reducing the nitro group to the corresponding amine as observed in case of *M. smegmatis* (34). In this regard, the results obtained from this study imply that increased expression of decaprenyl phosphate synthase (*uppS*) might provide an alternative mechanism for *Corynebacterineae*, to become resistant to BTZ. Furthermore, UppS might also serve as an effective drug target because its role is crucial in synthesis of a variety of mycobacterial cell wall components.

## Footnotes

3 The abbreviations used are:

TB: tuberculosis
MDR: multidrug resistant
XDR: extensively drug resistant
DPR: decaprenylphosphoryl-d-ribose
MIC: minimum inhibitory concentrations
DPA: decaprenylphosphoryl-d-arabinose
BHI: brain heart infusion
UppS: decaprenyl-phosphate synthase
IPP: isopentenyl pyrophosphate
DMSO: dimethyl sulfoxide
mAGP: mycolylarabinogalactan peoptidoglycan
LM: lipomannan
LAM: lipoarabinomannan.
